# Delayed Presentation of Giant Condyloma Acuminatum: A Case Report

**DOI:** 10.7759/cureus.106945

**Published:** 2026-04-13

**Authors:** Celia M Canova, Gabriela Del Sol, Amal Khallouki, Damian Casadesus

**Affiliations:** 1 Internal Medicine, Jackson Memorial Hospital, Miami, USA; 2 Medicine, St. George's University, St. George's, GRD

**Keywords:** buschke-löwenstein tumor, delayed diagnosis, giant condyloma acuminatum, human papilloma virus, social determinants of health

## Abstract

Buschke-Löwenstein tumor (BLT), or giant condyloma acuminatum, is a rare, slow-growing, locally invasive tumor associated with human papillomavirus (HPV). Although histologically benign, it demonstrates aggressive local growth, high recurrence rates, and potential for significant morbidity, necessitating timely diagnosis and surgical management. Risk factors include chronic HPV infection, immunosuppression, poor hygiene, and limited access to healthcare.

We report a 47-year-old man with a 15-year history of a progressively enlarging perianal mass, with delayed presentation due to embarrassment and financial barriers. The lesion extended circumferentially to the bilateral groin, scrotal, and perineal regions, with groin lesions measuring 12 × 6 × 6 cm on the left and 12 × 3 × 3 cm on the right, causing pain, malodor, and impaired mobility. Biopsy confirmed condyloma acuminatum without malignant transformation, with low-risk HPV positivity and wild-type p53 expression. The patient underwent staged surgical excision with multidisciplinary support, including social and financial services.

This case highlights the impact of social and economic barriers on delayed care, allowing otherwise manageable HPV-related lesions to progress to advanced disease. Early recognition and improved access to care are essential to prevent severe morbidity associated with BLT.

## Introduction

Giant condyloma acuminatum, also referred to as Buschke-Löwenstein tumor (BLT), is a rare, locally aggressive tumor associated with chronic infection by human papillomavirus (HPV), most commonly low-risk genotypes 6 and 11 [1]. These tumors have been associated with immunosuppression, including in patients with HIV/AIDS or other immune defects, although they can occur in immunocompetent individuals [2]. The lesions characteristically involve the anogenital mucosa and perineal region, where they may present as flat, papular, or exophytic growths [1]. Clinical manifestations range from asymptomatic lesions to those associated with pruritus, pain, or local discomfort, as was seen in this patient [3]. Diagnosis is primarily established through clinical evaluation, although histopathological confirmation may be warranted in atypical presentations or when malignant transformation is suspected [1].

Therapeutic options for limited disease include topical pharmacologic agents or ablative modalities such as cryotherapy [1]. In contrast, extensive or refractory lesions may necessitate surgical excision or other advanced interventions [1]. By presenting this case, we aim to raise awareness about the consequences of delaying medical care and highlight the role that social determinants and lack of care play in the complications and prognosis of this disease.

## Case presentation

A 47-year-old African American man with a 15-year history of progressive BLT presented to the emergency department on September 14, 2025, with active bleeding, malodorous discharge, and severe groin pain. The lesions had progressively enlarged over more than a decade, extending from the bilateral groin to the perineal region, with recent spontaneous bleeding. His past medical history was notable for obesity (BMI 31) and a significant tobacco use history (2-3 packs per day for 25-30 years), with cessation in April 2025; he otherwise denied chronic medical conditions. He had received the Gardasil vaccination at age 45. The patient reported no sexual activity for the past 10 years due to embarrassment related to the lesions. He works in maintenance and construction. He had previously been scheduled for surgical excision but was unable to proceed due to lack of insurance coverage and financial constraints, contributing to delayed care.

On presentation, he was tachycardic and in distress due to pain. Laboratory workup revealed leukocytosis with left shift and normocytic anemia (hemoglobin 8 g/dL, decreased from 13 g/dL earlier in the year). Urinalysis was negative.

During hospitalization, the patient developed nocturnal fevers, chills, and worsening difficulty ambulating. His hemoglobin dropped to 6.8 g/dL on September 16, requiring transfusion of one unit of packed red blood cells (Table 1). Laboratory findings demonstrated a microcytic anemia, likely secondary to chronic blood loss from the friable lesions, as well as leukocytosis consistent with an underlying infectious or inflammatory process. Blood cultures were negative. In the setting of systemic symptoms and suspected groin cellulitis associated with extensive condylomatous disease, the patient was started empirically on intravenous vancomycin and metronidazole to provide broad-spectrum coverage, including gram-positive organisms such as methicillin-resistant *Staphylococcus aureus* and anaerobic pathogens commonly implicated in perineal soft tissue infections.

**Table 1:** Lab values on presentation and admission during the most recent hospitalization. MCV: mean corpuscular volume; MCH: mean corpuscular hemoglobin; MCHC: mean corpuscular hemoglobin concentration; RDW-CV: red cell distribution width-coefficient of variation

Hematology	Day 1	Day 3	Normal range
WBC count	14 ↑	14.1 ↑	4.0-10.5 × 10³/mcL
RBC count	3.3 ↓	2.82 ↓	4.20-5.60 × 10⁶/mcL
Hemoglobin	8 ↓	6.8 ↓	13.3-16.3 g/dL
Hematocrit	25.2 ↓	22.1 ↓	39.0%-47.1%
MCV	76.4 ↓	78.4 ↓	79.9-95.0 fL
MCH	24.2 ↓	24.1 ↓	27.1-33.1 pg
MCHC	31.7 ↓	30.8 ↓	32.2-36.5 g/dL
RDW-CV	15 ↑	15.5 ↑	11.0%-15.0%
Platelet	433 ↑	379	140-400 × 10³/mcL
Neutrophil	69.9	72.9 ↑	36.0%-70.0%
Lymphocyte	14.7 ↓	11.1 ↓	16%-43.0%
Absolute neutrophil	9.8 ↑	10.3 ↑	2.0-6.0 × 10³/mcL
Absolute monocyte	1.3 ↑	1.7 ↑	0.3-0.9 × 10³/mcL

**Figure 1:**
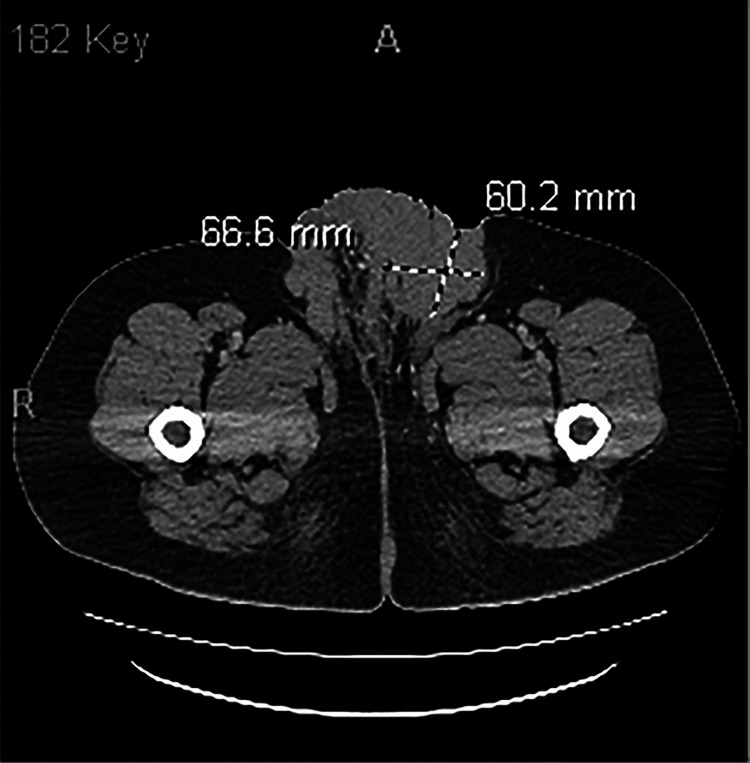
2025 CT abdomen and pelvis w/ intravenous (IV) contrast only (multiple volumetric CT images of the abdomen and pelvis were obtained from lung bases to pubic symphysis with IV contrast for evaluation for necrotizing fasciitis/hx of giant condyloma). Interval enlargement of coalescent fungating lesions of the bilateral groin, scrotum, and perineum with central gas foci (necrosis vs. skin folds), concerning for infiltrative neoplasm; superimposed infection not excluded. Minimal fluid along the left anteromedial thigh fascia without fascial gas. Extensive pelvic and inguinal lymphadenopathy.

**Figure 2:**
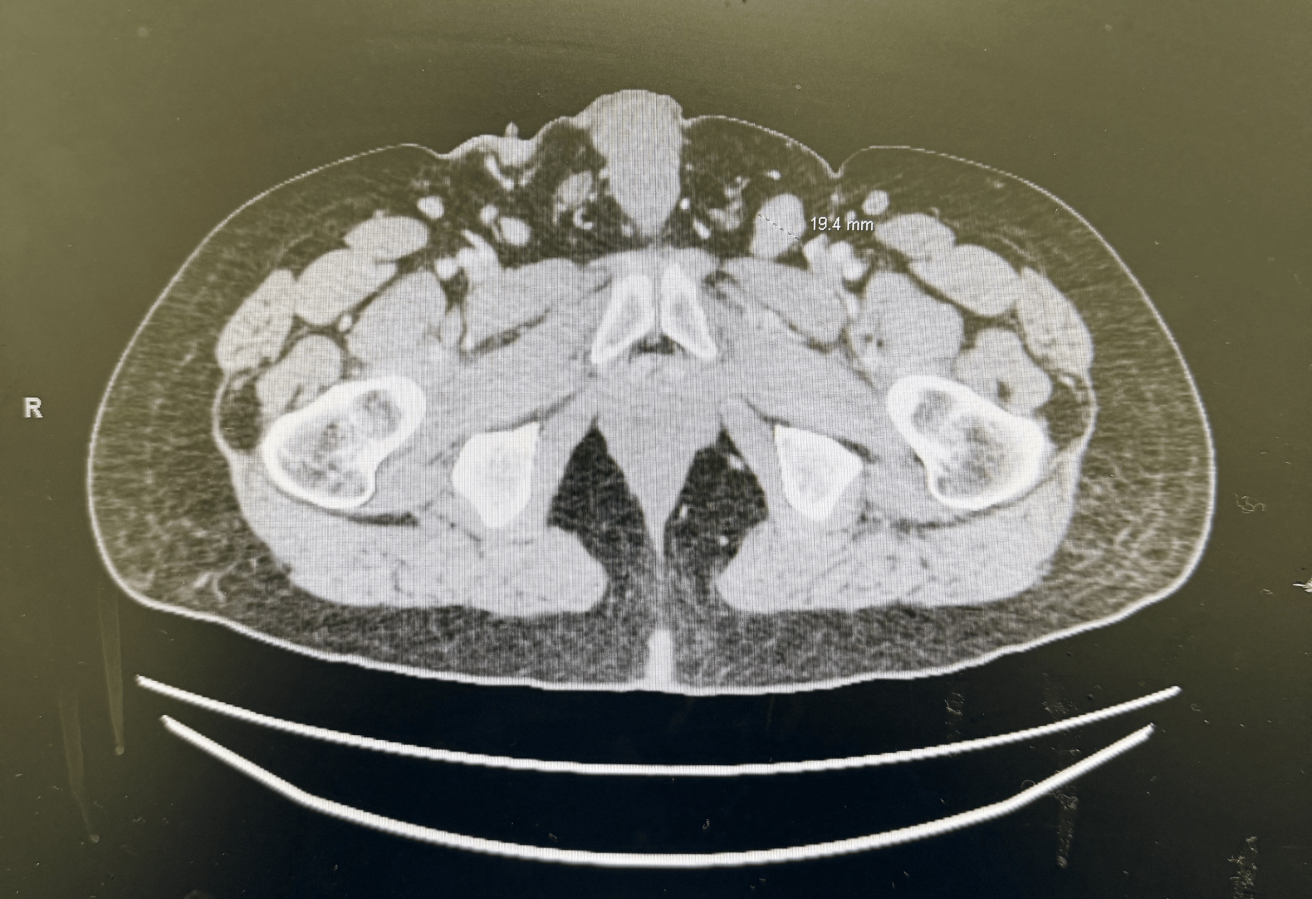
2024 CT pelvis with contrast (multiple axial CT images of the pelvis were obtained with intravenous (IV) contrast to evaluate numerous pelvic masses). Soft tissue thickening and fungating lesions are inseparable from the testicular tissues on this exam. There is a hypodense lesion within the left testicle measuring approximately 1.1 cm. Enlarged left inguinal lymph node measuring up to 1.9 cm and left pelvic sidewall lymph node measuring up to 1.6 cm.

CT of the abdomen and pelvis with contrast (September 15, 2025) demonstrated interval enlargement and coalescence of fungating lesions, measuring approximately 12 × 6 × 6 cm on the left and 12 × 3 × 3 cm on the right, involving the bilateral groin, scrotum, and perineum. Central gas foci were noted, possibly representing necrotic components (Figure 1). However, there was minimal fluid tracking along the left anteromedial thigh fascia, but no radiologic evidence of necrotizing fasciitis. Compared to a prior CT scan from March 2024, which did not include documented lesion measurements, the current imaging demonstrated clear interval progression in size and extent based on radiologic assessment (Figure 2). Extensive pelvic and inguinal lymphadenopathy was also observed in both of the 2024 (left inguinal lymph node measuring up to 1.9 cm) and 2025 (left inguinal lymph node measuring up to 2.1 cm) imaging. Of note, an ultrasound-guided left inguinal lymph node biopsy performed in November 2024 demonstrated paracortical hyperplasia with monoclonal B-cell hyperplasia, without immunophenotypically abnormal B- or T-cell populations, findings consistent with a reactive lymphoid process. Given these benign results, no further lymph node biopsy was pursued, including on the current 2025 imaging. Furthermore, a soft tissue ultrasound (performed on September 15, 2025) confirmed diffuse skin thickening with a soft tissue mass and no drainable collection.

**Figure 3:**
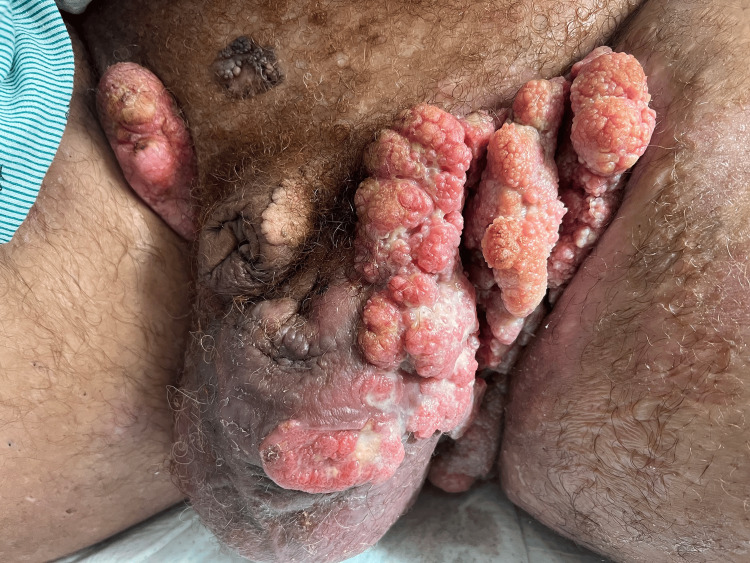
Image of lesions upon admission demonstrating large, verrucous, cauliflower-like masses involving both groin folds and extending into the perineal and scrotal regions. Written informed consent for publication of this image was obtained from the patient.

Examination upon arrival revealed large, verrucous, cauliflower-like masses involving both groin folds and extending into the perineal and scrotal regions (Figure 3). The lesions were intermittently bleeding and ulcerated with areas of malodor. Palpable, tender right inguinal lymphadenopathy was noted. The lesions significantly limited his ability to walk and perform daily activities.

The patient's medical history demonstrated recurrent challenges with these lesions. The patient underwent an examination under anesthesia and partial anal wart removal in August 2024, with pathology consistent with condyloma acuminatum, low-grade squamous intraepithelial lesion (LSIL), and anal intraepithelial neoplasia grade 1 (AIN1). A prior left groin lesion biopsy from March 2024 also confirmed condyloma acuminatum.

When further testing was done to check for any potential risk factors that may have precipitated his condition, screening for HIV, hepatitis B and C, syphilis, chlamydia, and gonorrhea were all negative. He had no history of immunosuppression.

The patient has an extensive history of hospitalizations due to the condyloma acuminatum. In March 2024, he presented to the emergency department with complaints of an enlarging groin, perineal, and anal masses that had been present for over 10 years, sometimes draining serous fluid and causing pain with ambulation. He was discharged with doxycycline and mupirocin and instructed to follow up with dermatology for a biopsy, which he was unable to complete due to financial limitations until August of that year. A prior visit to Mount Sinai Hospital yielded temporary improvement after antibiotic and analgesic therapy, but he received no biopsy or specialist referral.

**Figure 4:**
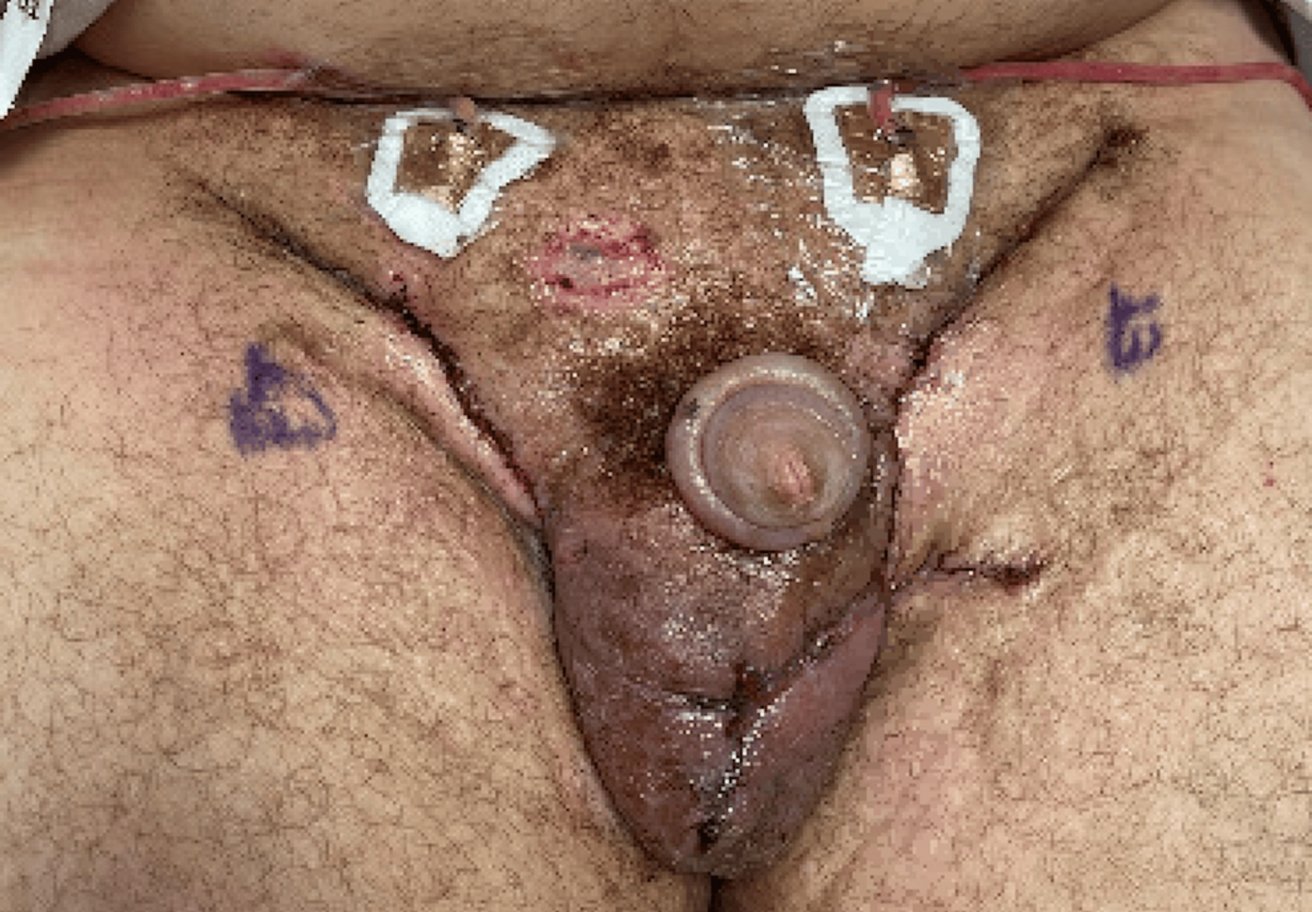
Postoperative image of reconstruction of condyloma acuminatum. Written informed consent for publication of this image was obtained from the patient.

The most recent hospitalization was crucial as he was managed with intravenous antibiotics, blood transfusion, and pain control while awaiting surgery. Since the September 15, 2025, admission, the hospitalization was prolonged while awaiting activation of insurance coverage necessary to proceed with definitive surgical intervention. On October 1, 2025, he underwent bilateral groin condyloma excision with complex closure of right inguinal, left inguinal, and scrotal wounds using adjacent tissue transfer. Urology and plastic surgery jointly reconstructed penile defects (Figure 4). The estimated total blood loss was 225 cc. Once all lesions were excised, complex, tension-free closure of all wound edges proceeded. However, the upper left inguinal wound edges could not be closed without tension, so a transposition flap was designed from the left medial thigh. Once all edges were closed, 219 French drains were placed bilaterally in the inguinal regions (one drain on each side). Furthermore, the pathology report from the excised bilateral inguinal and penile specimens demonstrated condylomas positive for low-risk HPV and negative for high-risk HPV. The epithelial cells showed a wild-type p53 immunoreactivity. Despite the 15-year duration, no focus of squamous cell carcinoma or any other malignancy was identified.

In the immediate postoperative period, the patient experienced mild pain that intensified to 9/10 by day three, described as burning and sharp in the perineal and scrotal regions, exacerbated by ambulation. The pain management service initiated oxycodone and hydromorphone, which effectively controlled his symptoms and allowed for gradual improvement in mobility. Upon discharge, he was prescribed celecoxib 100 mg twice daily, gabapentin 100 mg three times daily, acetaminophen 1,000 mg every eight hours, and oxycodone 5 mg as needed. He was also started on doxycycline and levofloxacin prophylactically. The patient was instructed to avoid heavy lifting, strenuous activity, and immersion in water for two months. Wound care consisted of Aquacel Ag (Convatec, Reading, UK) and bacitracin to incisions twice a day, followed by kerlix, and mesh underwear.

Both French drains reached a maximum serosanguineous (ss) output of 30-40 cc. On October 15, 2025, the right French drain was removed due to less than 10 cc of ss output. The left French drain was removed on October 29, 2025, once reaching less than 10 cc of ss output. By December 17, 2025, the patient was ambulating well with adequate pain control and was independently performing wound care, including showering at least three times daily. By January 2026, the patient reported complete resolution of pain and significant improvement in overall quality of life. However, by March 2026, the patient presented to the urology office with condylomatous lesions recurring bilaterally to the penile base measuring up to 5 mm in size. The plan was to perform excision of the condylomas.

This case illustrates how socioeconomic instability and psychosocial barriers can contribute to extreme disease progression [4]. The patient’s lack of insurance repeatedly delayed access to surgical intervention [5]. Embarrassment and stigma surrounding a genital condition further prevented timely medical care [6]. Despite multiple encounters with healthcare providers, he received only temporary symptom relief rather than definitive treatment. By the time he was able to obtain coverage, the lesions had become extensive, ulcerated, and infected, culminating in anemia and sepsis [4]. His case underscores the importance of addressing financial and social determinants of health to prevent avoidable morbidity from otherwise treatable conditions [7].

## Discussion

BLT is a rare, slow-growing, locally invasive neoplasm associated with HPV, most commonly types 6 and 11. Although histologically benign, BLT demonstrates aggressive local behavior, with reported malignant transformation rates ranging from approximately 30% to 56% in longstanding or untreated cases [1,2,8,9]. The current standard of care is wide surgical excision with negative margins, as conservative or medical therapies alone are associated with high failure and recurrence rates [1,2].

This case is notable for two key features: the extraordinary delay in presentation and the significant impact of socioeconomic barriers on disease progression. The patient lived with a progressively enlarging genital tumor for over 15 years prior to definitive treatment, representing an extreme duration rarely described in the literature. This prolonged delay allowed for the development of massive bilateral lesions involving the groin, scrotum, and perineum, ultimately requiring extensive surgical management. While delayed presentation has been reported in BLT, the magnitude and duration observed in this case are particularly striking.

From a clinical standpoint, early surgical intervention is critical in preventing complications such as local tissue destruction, secondary infection, anemia, and sepsis. In this patient, delayed care resulted in advanced disease requiring complex management. Wide local excision remains the cornerstone of treatment, and timely intervention has been associated with improved outcomes and decreased morbidity [1,2]. In contrast, delayed or incomplete treatment contributes to higher recurrence rates, which have been reported to range from approximately 20% to 67% in the literature [1-3].

Despite appropriate surgical management, BLT carries a substantial risk of recurrence, necessitating close postoperative surveillance. Compared to these reported outcomes, our patient demonstrated a favorable early postoperative course, with complete resolution of pain and significant improvement in quality of life within one month following surgery. However, long-term follow-up remains essential to monitor for recurrence or malignant transformation.

This case also underscores the profound role of socioeconomic determinants of health in shaping clinical outcomes. The patient’s delay in seeking and maintaining care was primarily driven by lack of medical insurance, financial hardship, and stigma associated with the genital location of the disease [5,10]. Despite multiple healthcare encounters, he received only temporary symptomatic management rather than definitive surgical intervention. These gaps in care highlight systemic barriers that continue to limit access to specialty services for vulnerable populations [11].

Stigma surrounding sexually transmitted infections (STIs) further contributed to delayed presentation. Feelings of embarrassment, social isolation, and fear of judgment are well-documented factors that reduce healthcare utilization in patients with genital conditions [10]. In this case, these psychosocial factors compounded existing financial barriers, resulting in prolonged, untreated disease and significant morbidity.

Addressing such disparities requires a multidisciplinary approach that extends beyond surgical management. Integration of social support services, improved access to specialty care, and targeted public health interventions are essential to bridge gaps in care. Preventive strategies, including HPV vaccination and education aimed at reducing STI-related stigma, remain critical in mitigating the progression of BLT to advanced stages [12].

In summary, this case highlights a rare presentation of BLT characterized by extreme delay in treatment and significant socioeconomic barriers leading to advanced bilateral disease. It emphasizes the importance of early surgical intervention, vigilant postoperative follow-up, and addressing social determinants of health to improve outcomes in patients with this condition.

## Conclusions

This case describes a 47-year-old man with no known predisposing conditions who presented with extensive condyloma acuminatum of the groin and perineum, progressing over more than 15 years. It highlights an unusually prolonged disease course resulting in advanced bilateral involvement requiring definitive surgical excision.

The case underscores the significant impact of socioeconomic and psychosocial barriers-including lack of insurance, limited access to care, and stigma-on delayed diagnosis and treatment, ultimately contributing to severe disease progression. Although the patient experienced marked symptomatic improvement following surgery, the risk of recurrence remains, and close long-term follow-up is essential. This case emphasizes the importance of early recognition, timely surgical intervention, and addressing social determinants of health to prevent advanced presentations.
